# Cortico-Cerebellar Connectivity in Autism Spectrum Disorder: What Do We Know So Far?

**DOI:** 10.3389/fpsyt.2016.00020

**Published:** 2016-02-23

**Authors:** Alessandro Crippa, Giuseppe Del Vecchio, Silvia Busti Ceccarelli, Maria Nobile, Filippo Arrigoni, Paolo Brambilla

**Affiliations:** ^1^Scientific Institute, IRCCS Eugenio Medea, Lecco, Italy; ^2^Department of Psychology, University of Milano – Bicocca, Milan, Italy; ^3^Department of Clinical Neurosciences, Hermanas Hospitalarias, FoRiPsi, Albese con Cassano, Italy; ^4^Department of Neurosciences and Mental Health, Fondazione IRCCS Ca’ Granda Ospedale Maggiore Policlinico, University of Milan, Milan, Italy; ^5^Department of Psychiatry and Behavioral Sciences, University of Texas Health Science Center at Houston, Houston, TX, USA

**Keywords:** cortico-cerebellar connectivity, autism spectrum disorders, autism, DTI, fMRI, resting-state fMRI

## Abstract

Although the Autism Spectrum Disorder (ASD) is renowned to be a connectivity disorder and a condition characterized by cerebellar involvement, the connectivity between the cerebellum and other cortical brain regions is particularly underexamined. Indeed, converging evidence has recently suggested that the cerebellum could play a key role in the etiopathogenesis of ASD, since cerebellar anomalies have been consistently reported in ASD from the molecular to the behavioral level, and damage to the cerebellum early in development has been linked with signs of autistic features. In addition, current data have shown that the cerebellum is a key structure not only for sensory-motor control, but also for “higher functions,” such as social cognition and emotion, through its extensive connections with cortical areas. The disruption of these circuits could be implicated in the wide range of autistic symptoms that the term “spectrum” connotes. In this review, we present and discuss the recent findings from imaging studies that investigated cortico-cerebellar connectivity in people with ASD. The literature is still too limited to allow for definitive conclusions; however, this brief review reveals substantial areas for future studies, underlining currently unmet research perspectives.

## Introduction

Autism spectrum disorder (ASD) is a multifaceted neurodevelopmental disorder characterized by persistent social impairment, communication abnormalities, and restricted and repetitive behaviors (DSM-5) (1, 2). ASD is a complex condition with an average prevalence of about 1% worldwide (3), one in 68 U.S. children (4). Although high heritability estimates suggest a critical role for genetic factors (5), its etiology is generally considered multifactorial. It has been hypothesized that the heterogeneous phenotype of ASD could implicate a greater likelihood of abnormalities in the connectivity between different neural networks rather than alterations in a specific cerebral area (6). Over the last decade, the claim that ASD is a disorder of connectivity has been reliably supported by evidence from neuroimaging studies (7, 8), even though with mixed findings. On one hand, some studies have provided initial evidence of underconnectivity in ASD (9–11); on the other hand, another line of research has indicated overconnectivity in ASD, arguing for an increased local and short-distance connectivity within the frontal cortex, with respect to reduced long-range connectivity between frontal lobes and posterior brain regions (12–14).

Two recent studies (15, 16) that analyzed the database of fMRI resting-state scans from the Autism Brain Imaging Data Exchange have revealed the occurrence of underconnectivity and overconnectivity in ASD, although with different topographical distributions. More precisely, overconnectivity seems to be primarily associated with subcortical regions, whereas hypoconnectivity appears to characterize the pattern of cortico-cortical and interhemispheric functional connectivity.

However, the connectivity between different brain areas with the cerebellum is still a particularly under-considered issue in ASD research. Although it was traditionally believed that the cerebellum was exclusively a motor structure (17), converging evidence suggested a role for the cerebellum in other “higher” functions, including language and cognition, as well as emotion (18–20). Indeed, neuroanatomical findings have clearly shown that the cerebellum can influence a number of neocortical areas, including premotor, prefrontal, and posterior parietal areas of the cerebral cortex, through polysynaptic circuits via thalamus and basal ganglia. These pathways subserved specifically different functions, such as movement, cognition, and social skills (21–24). On the basis of decades of anatomical and imaging data [see Ref. (25, 26, 27–30) for reviews], it has been suggested that the cerebellum could be primarily implicated in ASD (31, 32), with cognitive and behavioral effects beyond the difficulties in the motor domain (33, 34). In fact, a cerebellar dysfunction early in development has been associated with deficits in executive functions, visual-spatial processing, linguistic function, and affective regulation (35, 36), and even with social difficulties, such as avoidance of physical contact or gaze aversion, within a diagnosis of ASD (37). Moreover, Wang et al. (36) recently proposed that cerebellar damage in childhood may perturb the maturation of distant neocortical circuits during developmental sensitive periods through a “developmental diaschisis,” increasing the risk for developing ASD.

Despite its connections with several brain areas and the well-known involvement of this structure in the disorder, few imaging studies have investigated the role of cerebro-cerebellar connections in ASD and correlations between cerebro-cerebellar connectivity and clinical measures. Considering this lack of evidence, we briefly summarized the recent imaging findings on cortico-cerebellar connectivity in ASD in order to (a) address strengths and pitfalls of previous studies and (b) explore potential strategies for future research. In addition, we aimed to understand whether disruptions of specific cortico-cerebellar circuits could be associated with specific difficulties in ASD or different phenotypes within the disorder. Publications for this review were identified from a PubMed search in November 2015 using terms related to autism, connectivity, magnetic resonance (MR) imaging, and cerebellum. This search was supplemented with other publications from the reference lists of all included citations, and from the personal reference databases of the authors.

## Diffusion Imaging Studies

Structural connectivity can be assessed *in vivo* using MR techniques like diffusion-weighted imaging (DWI) or Diffusion Tensor Imaging (DTI). These non-invasive techniques provide indirect quantitative measures of white matter integrity, such as fractional anisotropy (FA), mean diffusivity, axial diffusivity, and radial diffusivity, by measuring water diffusion in the underlying tissue microstructure (38). Mean diffusivity is the average of the diffusion in the different directions of the space, and its values are related to the presence of barriers or obstacles, like cellular membranes and axons, which can interfere with the free water displacement within a voxel. When diffusion of water molecules is not the same along the three axes of the space (as in axons), it is called anisotropic, which means it has a preferential direction of displacement. Axial and radial diffusivity measure the entity of displacement along the principal and its perpendicular axis. FA values, which range between 0 and 1, are also a measure of anisotropy that seem to be related with myelination, axon diameter, and fiber coherence (39). High FA values denote well organized and normally myelinated axons that provide natural barriers to water movement within tissue. Lower FA values, in contrast, may reflect axonal loss and/or demyelination (39) as well as areas of crossing fibers. DWI allows for quantification of FA at voxel levels, whereas DTI, using different tracking algorithms, enables reconstruction of structural connections. An overview of the studies on structural connectivity between the cerebellum and different cerebral areas in ASD can be found in Table 1 (40–45). When using the terms overconnectivity or underconnectivity in diffusion imaging studies, we refer here to connectivity disruption in terms of tissue organization.

**Table 1:** **Diffusion imaging studies investigating cerebro-cerebellar connectivity in ASD**.

Study	Participants (*N*, age range)	Methods	Findings	Relationship connectivity measures – behavior
Catani et al. (40)	15 Asperger, 16 HC, 18–49 years	DTI–ROI	↓ FA in the right superior cerebellar peduncle and in the right short intracerebellar fibers	No differences in the mean diffusivity	Negative correlation between the ADI-R social domain score and FA of the left superior cerebellar peduncle
Brito et al. (41)	Eight with autism, eight HC, 6–12 years	DTI–ROI	↓ FA in the left superior cerebellar peduncle, and in the right and in the left middle cerebellar peduncles	NA
Shukla et al. (42)	26 ASD, 24 HC, 9–18 years	DTI–ROI	↓ FA in the middle cerebellar peduncle	No differences in the mean diffusivity, axial or radial diffusion	No correlations with ADI or ADOS scores
Sivaswamy et al. (43)	27 ASD, 16 HC, 2.6–9 years	DTI–ROI	↑ Mean diffusivity of the bilateral superior cerebellar peduncles	NA
			↑ FA in the right middle cerebellar peduncle	
			Reversed pattern of asymmetry in the FA of the middle and inferior cerebellar peduncles	
Hanaie et al. (44)	13 ASD, 11 HC, 5–14 years	DTI–ROI	↓ FA in the right superior cerebellar peduncle	Positive correlation between the M-ABC 2 total score and FA in the right superior cerebellar peduncle
			↓ Axial diffusivity in the left superior cerebellar peduncle	
Jeong et al. (45)	15 ASD, 14 HC, 3.6–13	DWI – ICA + BSM tractography	↓ Streamline volume and count between cerebellar cortex and the right VDN	Positive correlation between FA of right dorsal dentate nucleus and VABS 2 – daily living skills
			↓ FA between cerebellar cortex and the right DDN, and VDN bilaterally	
			↓ Axial diffusivity between cerebellar cortex and the left DDN, and left VDN	

*ASD, autism spectrum disorder; HC, healthy controls; DTI, diffusion tensor imaging; DWI, diffusion-weighted imaging; ICA + BSM, independent component analysis with a ball and stick model; ROI, region of interest; FA, fractional anisotropy; VDN, ventral dentate nucleus; DDN, dorsal dentate nucleus; NA, not assessed; ADI-R, Autism Diagnostic Interview-revised; M-ABC 2, Movement Assessment Battery for Children – second edition; VABS, Vineland Adaptive Behavioral Scales*.

The majority of results from diffusion imaging studies showed a weaker structural connectivity in participants with ASD, as indicated by decreased FA both in the superior cerebellar peduncles (40, 41, 44) and in intracerebellar circuitries (45). However, findings in the middle cerebellar peduncles did not yield consistent evidence. Shukla et al. (42) revealed reduced values of FA in adolescents with ASD; conversely, Sivaswamy et al. (43) found increased values of FA in the right middle cerebellar peduncle, although within a reverse asymmetry pattern in FA of the middle and inferior cerebellar peduncles. A quantitative tractography study (45), in which a newly developed method for DWI called the “independent component analysis with a ball and stick model” was used to reveal abnormally reduced volume and number of fibers between the cerebellar cortex and right ventral dentate nucleus, accompanied by decreased FA between the cerebellar cortex, right dorsal dentate nucleus, and bilateral ventral dentate nucleus. Alterations of FA in cerebellar structures are mainly, but not always, reported to occur in association with reduced axial diffusivity [Ref. (44, 45); but not Ref. (42)], in absence of abnormalities of mean or radial diffusion.

In respect to the relationship between white matter integrity, behavior, and ASD symptoms, Catani et al. (40) found a negative correlation between FA values in the right superior cerebellar peduncle and in the right short intracerebellar fibers, in addition to social difficulties as reported by a caregiver using ADI-R (46), a “gold standard” diagnostic interview tool for clinical diagnosis. Moreover, Jeong et al. (45) depicted a relation between lower FA values between the cerebellar cortex and the right dorsal dentate nucleus, and between the ventral dentate nucleus bilaterally and poor daily living skills measured by Vineland Adaptive Behavioral Scales (47). Lastly, the motor abilities measured at Movement ABC-2 (48) were found to be positively correlated to FA in the right superior cerebellar peduncle (44). However, Shukla et al. (42) reported no relationship between DTI measures and clinical symptoms of ASD.

In sum, findings from diffusion imaging studies indicate underconnectivity – in reference to a different white matter integrity and coherence between participants – between the cerebellar main outflow pathways (i.e., the superior cerebellar peduncles) and the neocortex, and in intracerebellar circuitries that involve the dentate nucleus. Results for the middle and inferior cerebellar peduncles are not so consistent, with mixed reports of reduced and increased structural connectivity. Nevertheless, these findings altogether seem to suggest a possible abnormal connectivity between the cortical areas and the main afferent fibers of the cerebellum. Finally, the findings from the reviewed studies suggested some preliminary evidence of a relationship between the structural connectivity of the cerebellum and manifestations of ASD.

## Task-Related Functional Imaging Studies

Functional brain connectivity can be effectively quantified during both task performance and rest by correlating variations of the blood-oxygen-level-dependent (BOLD) signal over time (49, 50). Different neuroanatomical regions are assumed to be functionally connected when the time courses of the BOLD fluctuations in these regions have synchronized patterns of activation (51). To the best of our knowledge, only three studies investigated the functional connectivity between cerebellum and cortical areas during task performance in ASD. Mostofsky et al. (52) assessed activation during a sequential finger tapping task in 13 children with high-functioning autism aged 8–12 years and in 13 age-matched typically developing peers, using functional magnetic resonance imaging (fMRI). The authors found activations in motor circuits across participants, which include contralateral pre/postcentral gyrus, ipsilateral anterior cerebellum (lobules IV/V), bilateral activation in the superior medial wall (BA6), and contralateral activation in the thalamus. However, children with typical development showed recruitment of cerebellar structure, i.e., the anterior lobe of the contralateral cerebellum (lobules IV/V) and ipsilateral anterior cerebellum, that is absent in autistic children. Conversely, the clinical group showed an increased activation of the supplementary motor area. In addition, a reduced functional connectivity within the motor circuits including premotor areas and the cerebellum was observed in autistic children, suggesting alterations in long-range connections in the fronto-cerebello-thalamo-frontal network.

Jack and Morris (53) directly investigated the relationship between functional connectivity and ASD features in an fMRI study on the neural bases of perception and use of human actions in imitation. Using psychophysiological interaction (PPI) analysis, the authors indicated an involvement of the network between posterior superior temporal sulcus (pSTS) – neocerebellum (i.e., Crus I) in social cognition in both adolescents with and without ASD. Although PPI data did not differ between groups, the authors showed that functional coactivation of pSTS and Crus I could predict the social deficits in ASD, as rated by parents on a questionnaire assessing “mentalizing skills,” (54) i.e., the ability to attribute mental states to others.

Recently, Kana et al. (55) examined the neural network underlying theory of mind, including the cerebellum, in high-functioning children and adolescents with autism while they were decoding the interactions between animated figures. The authors found a reduced cerebellar activation, particularly in Crus I, in participants with ASD in the theory of mind condition. Furthermore, they outlined reduced functional connectivity in ASD between the cerebellum and medial regions (i.e., medial prefrontal cortex and posterior cingulate cortex).

## Resting-State Imaging Studies

Five recent studies that use resting state to assess cerebro-cerebellar connectivity were included in the present review (see Table 2 for an overview).

**Table 2:** **Resting-state imaging studies investigating cerebro-cerebellar connectivity in ASD**.

Study	Participants (*N*, age range)	Methods	Findings	Relationship connectivity measures – behavior
Padmanabhan et al. (56)	42 ASD, 48 HC, 8–36 years	ROI (striatal seed regions)	Different developmental trajectory of FC among striatum, cerebellar lobules VI and VIIa, and Crus I	No correlations with ADI-R score
Verly et al. (57)	19 ASD + LI, 23 HC, mean age (SD): 14.3 years (1.3), 14.0 years (1.5)	ROI (seed regions) plus voxel-based analysis	↓ FC within the cerebello-DLPF, cerebello-SMA, cerebello-IFG, and cerebello-premotor circuits	Negative correlation between the ASD severity factor and FC between the right cerebellum and left DLPF seed
Khan et al. (58)	28 ASD, 28 HC, 8–17 years	ROI	↑ Overall FC between cerebrum and cerebellum	No correlations with ADI-R
			↑ FC between cortical regions of one domain (motor or supramodal) and cerebellar regions of the other	Negative correlation between the cerebellar FC with the right supramodal ROI and non-verbal IQ
			↑ FC for the sensorimotor ROIs but ↓ FC for the supramodal ROIs	
Carper et al. (59)	44 ASD, 36 HC, 7–18 years	Seed regions	No group differences in FC between cerebellum and M1	No correlations between FC and clinical symptoms after correction for multiple comparisons
Dajani and Uddin (60)	53 ASD, 53 HC, three stratified groups: children <11 years, adolescents 11–18 years, adults ≥18 years [data from ABIDE (14)]	Regional Homogeneity (ReHo)	Children: ↓ ReHo in cerebellar lobule VI	Positive correlation between mean ReHo values and SCQ
			Adolescents: ↑ ReHo in cerebellar lobule IX	
			Adults: ↓ ReHo in cerebellar vermis, bilateral lobule VI, and Crus I	

*ASD, autism spectrum disorder; LI, language impairment; HC, healthy controls; ROI, region of interest; FC, functional connectivity; DLPF, dorsolateral prefrontal; SMA, supplementary motor area; IFG, inferior frontal gyrus; ADI-R, Autism Diagnostic Interview-revised; SCQ, Social Communication Questionnaire*.

Verly et al. (57) investigated the role of the cerebellum and its functional connectivity in the classic areas of the language network in children with ASD and language impairment. To do this, a verb generation fMRI task was first used to define language areas commonly active in participants with and without ASD. Afterward, the selected regions were used as seeds for resting-state analysis, in addition to the traditional voxel-based analysis. Results from both the seed-based and voxel-wise maps indicated a significantly reduced functional connectivity in ASD among cerebellum, Broca’s, and Wernicke’s areas. The authors interpreted this dissociation of cerebral and cerebellar language regions as a possible index of altered cerebellar modulation of language functioning.

The study by Khan et al. (58) is, to date, the first work that aimed to directly assess the cerebro-cerebellar connectivity in ASD. The authors used resting-state MRI to measure the functional connectivity between the cerebellum and seven bilateral cortical regions of interest (ROIs) in 28 children and adolescents with ASD compared to their typically developing peers. Cerebral regions were grouped in sensorimotor ROIs (premotor and primary motor cortices, somatosensory superior temporal cortex, and occipital lobe) and in supramodal ROIs (prefrontal cortex, posterior parietal cortex, and inferior and middle temporal gyri). Overall, the authors found a general cerebro-cerebellar overconnectivity in the ASD group. In addition, the analysis of the connections’ domain-specificities revealed an increase in non-canonical links, i.e., in the connections between cortical regions of one domain (sensorimotor or supramodal) and cerebellar regions of the other. Furthermore, an increased cerebro-cerebellar connectivity was also found in sensorimotor circuitries at the expense of connectivity in supramodal “cognitive” networks (reduced in ASD).

Carper et al. (59) have recently investigated the anatomical and functional connectivity of the motor control system in children and adolescents with ASD compared to healthy controls. With regard to the connectivity between the cerebellum and M1, the authors did not find any group differences in functional connectivity.

Finally, the other two resting-state studies reviewed here aimed to assess the functional connectivity of the cerebellum across development (56, 60). Indeed, both studies were cross-sectional and recruited participants of different ages, from childhood to adulthood. Findings from these works consistently indicated abnormal developmental trajectories of functional connectivity. Specifically, Padmanabhan et al. (56) found an increase of connectivity over development between cerebellar and subcortical regions (i.e., the striatum nucleus) in people with ASD, but a decrease in healthy controls. Dajani and Uddin (60) used regional homogeneity (ReHo) analysis to individuate local patterns of connectivity within the cerebellum. The authors were able to describe an age-specific pattern of short-range connectivity in ASD, with children and adults having lower ReHo in the cerebellum than controls, while adolescents exhibited an increased cerebellar local connectivity.

In respect to the relationship between functional connectivity and ASD features, findings from the study analyzed here are not entirely consistent. Indeed, no links between connectivity and “gold standard” clinical measures of ASD were observed (56, 58), although reduced connectivity seem to be accompanied by an increase in the severity of the disorder (57, 60), as assessed by the Social Communication Questionnaire [SCQ, Ref. (61)]. Lastly, lower values of connectivity between cerebellum and supramodal “cognitive” areas have been observed to be linked to higher non-verbal IQ (58).

## Summary and Future Directions

In the present work, we aimed to provide an up-to-date overview of current findings on cortico-cerebellar connectivity in ASD. This issue represents an emerging field of interest for ASD research, following the hypothesis that ASD is a connectivity disorder associated with cerebellar dysfunctions. The cerebellum has been recently indicated as a key structure not only for sensory-motor control, but also for language, social cognition, and emotion, via its extensive connections with cortical areas (33–37). Although the literature is at a very early stage and more work on cortico-cerebellar connectivity is urgently needed, some preliminary suggestions can be drawn from the reviewed studies. Findings from task-related imaging studies showed a pattern of underconnectivity between the cerebellar outflow pathways and the neocortex, and in long-range fronto-cerebello-thalamo connections. Results from diffusion imaging studies are partly in line with these conclusions, although it is worth noting that this technique does not provide any direct measure of connectivity but is solely an index of fibers coherence and integrity. Significantly reduced long-range functional connectivity, among cerebral and cerebellar language regions, was also found in a resting-state study. However, results from afferent fibers of the cerebellum and from other resting-state studies indicated more complex, or even opposite, patterns of findings, disallowing any firm conclusion at this time. The causes of this discrepancy might be various, as the studies differed in many important methodological aspects. As clearly shown by Nair et al. (62), factors such as the type of analysis, the choice of seed placement, and the type of dataset can have a dramatic impact on results of functional connectivity studies in ASD. Keeping this in mind, a possible theoretical explanation could be a concurrence of under connectivity and overconnectivity between cortical areas and cerebellum. This suggestion seems to be supported by findings from a study that, for the first time, explicitly assessed cortico-cerebellar connectivity in ASD (58). Another explication of the partly conflicting reports may be the developmental changes in functional connectivity (63). Theoretically, this opinion is based on the account of ASD as a “developmental disconnection syndrome,” first proposed by Geschwind and Levitt, and more recently, by Wang et al. (36, 64). Given the developmental nature of the disorder, the connectivity abnormalities in ASD could vary in direction and in degrees of alteration through different stages of development as a result of neural plasticity. This hypothesis has received empirical support from diffusion imaging studies (65), resting-state studies using fMRI (66), and near-infrared spectroscopy (67). Abnormal developmental trajectories in ASD have been also found for the cortico-cerebellar connectivity (56, 60), as discussed above. Cross-sectional and longitudinal studies are warranted to control for the impact of studies’ differences in age ranges of participants on findings. In order to better understand possible developmental abnormalities of cortico-cerebellum connectivity, animal models can also provide useful insights into how a damage in cortico-cerebellar connections at a specific age could result in abnormal autistic-like behaviors (68).

Another area of concern raised by the evidence reviewed here is the lack of a specific relationship between connectivity and behavioral/diagnostic measures of ASD. This might be due, at least in part, to the well-known heterogeneity of people with ASD. Thus, with respect to the aim of our work, it is not possible at this stage to draw a direct link between the disruption of a specific cerebro-cerebellar circuit and a restricted behavioral phenotype of patients. Future research could overcome this limitation by including subsets of patients defined on the basis of different quantifiable measures of ASD phenotype, such as motor impairments, stereotyped behaviors, or language difficulties, in order to understand the relationship between these “proxy markers” of the disorder and the cortico-cerebellar connectivity. Within this context, more neuroimaging observations are also needed to localize ASD abnormalities in connectivity to specific areas of the cerebellum. To do this, it could be useful to couple both structural and functional imaging with experimental neurobehavioral paradigms that encompass the role of the cerebellum in movement, language, and social cognition.

## Author Contributions

AC and PB conceived, designed, acquired background material, and drafted this work; GD, SBC, MN, and FA interpreted the background material, critically revised, and approved the final version of and agreed to be accountable for this work.

## Conflict of Interest Statement

The authors declare that the research was conducted in the absence of any commercial or financial relationships that could be construed as a potential conflict of interest.
